# Raman Microscopy: Progress in Research on Cancer Cell Sensing

**DOI:** 10.3390/s20195525

**Published:** 2020-09-27

**Authors:** Satheeshkumar Elumalai, Stefano Managó, Anna Chiara De Luca

**Affiliations:** Institute of Biochemistry and Cell Biology (IBBC), National Research Council of Italy (CNR), Via P. Castellino 111, 80131 Naples, Italy; satheesh@ibbc.cnr.it (S.E.); stefano.manago@ibbc.cnr.it (S.M.)

**Keywords:** Raman spectroscopy, cell sensing, leukemia, breast cancer cell, Raman imaging, correlative imaging

## Abstract

In the last decade, Raman Spectroscopy (RS) was demonstrated to be a label-free, non-invasive and non-destructive optical spectroscopy allowing the improvement in diagnostic accuracy in cancer and analytical assessment for cell sensing. This review discusses how Raman spectra can lead to a deeper molecular understanding of the biochemical changes in cancer cells in comparison to non-cancer cells, analyzing two key examples, leukemia and breast cancer. The reported Raman results provide information on cancer progression and allow the identification, classification, and follow-up after chemotherapy treatments of the cancer cells from the liquid biopsy. The key obstacles for RS applications in cancer cell diagnosis, including quality, objectivity, number of cells and velocity of the analysis, are considered. The use of multivariant analysis, such as principal component analysis (PCA) and linear discriminate analysis (LDA), for an automatic and objective assessment without any specialized knowledge of spectroscopy is presented. Raman imaging for cancer cell mapping is shown and its advantages for routine clinical pathology practice and live cell imaging, compared to single-point spectral analysis, are debated. Additionally, the combination of RS with microfluidic devices and high-throughput screening for improving the velocity and the number of cells analyzed are also discussed. Finally, the combination of the Raman microscopy (RM) with other imaging modalities, for complete visualization and characterization of the cells, is described.

## 1. Introduction

Raman scattering, discovered by Sir C.V. Raman and K.S. Krishnan in 1928, refers to the scattering of light from a molecular or cellular sample that exhibits a frequency shift (inelastic scattering). The resulting energy difference between the incident photon and the Raman scattered photon, defined as the Raman shift (or) wavenumbers expressed as cm^−1^, corresponds to the energy of specific molecular vibrations within the sample of interest [1]. In this manner, Raman spectroscopy (RS) provides a detailed chemical composition of the sample—a chemical fingerprint in essence. The basic selection rule for observing the Raman scattering is that the polarizability of the molecules must change during vibrations by incident light [2]. The Raman intensity depends on the intensity of the laser source as well as the polarizability and concentration of the molecules in the samples [3]. This technique has enormous potential in the field of biomedical science, as it can be applied to samples over a wide size range, from single cells to intact tissues. Despite the promising applications, a major challenge in RS is the inherently weak nature of the signal. Indeed, a small fraction of the incident light undergoes Raman scattering, i.e., less than 1 in 10^6^ to 10^8^ of incident photons, while a large fraction is elastically scattered (Rayleigh scattering).

Recently, RS has garnered attention as a non-invasive technique owing to its ability to specifically identify biomolecules and its sensitivity to correctly providing diagnostic information to the clinician on the alteration of molecular signatures in a cell or tissue, as it does not require any histochemical staining [4]. Indeed, RS, detecting the fundamental vibrational states of biomolecules, is exploited as a label-free, non-invasive tool for monitoring the biochemical changes between normal and cancer cells [5]. Based on Raman spectral profile, differences in the composition of nucleic acids, proteins, lipids, and carbohydrates in cancer/normal cells helps in the evaluation, characterization, and discrimination of cancer stage [6,7,8,9].

Moreover, by coupling an optical microscope with RS, the so-called Raman microscope, allows the mapping and reconstruction of the morpho-chemical properties of analyzed sample, in a non-destructive and non-invasive fashion. On a different note, Raman imaging can overcome problems resulting from limited stability, bleaching, the use of external biomarkers and long sample preparation associated with traditional morphological analysis like electron microscopy and fluorescence microscopy, opening the way to in vivo analysis. Raman microscopy (RM) can be a complement to conventional staining methods that can be easily used for monitoring the sub-cellular components of normal and cancer cells [10,11]. Therefore, the application of RM can be used as a non-invasive method for the early diagnosis of cancer cells.

In this review, we show the RS-based imaging technique, and provide biochemical identification and mapping of normal and cancer cells. We choose two-examples, i.e., leukemia and breast cancer cells, as model systems to emphasis the advantages of RS and RM-based analysis for identification of cancer cells, classification and follow-up after chemotherapy treatments. We also discuss the quality, objectivity, speed and sampling capacity of the RS-based cell sensing. We introduce the importance of an automated and objective assessment of cancer cell diagnosis, showing the use of multivariate analyses, such as PCA/LDA, for Raman data managing. Finally, correlative imaging methods combining RM with other microscopies, such as optical coherence tomography (OCT), holography, fluorescence microscopy and mass-spectroscopy-based imaging, for a full understanding of the morphology and cell biochemistry, are described.

## 2. Discussions

### 2.1. Raman Spectroscopy for the Biochemical Identification of Cells

The diagnosis and grading of cancer are crucial for the choice of appropriate therapeutic protocol and significantly impact patient survival. Cancer cell and tissue samples are generally analyzed by H&E staining as a standard method for a preliminary morphological diagnosis, which is often used by pathologists. However, this can be achieved only by cellular staining and it shows low sensitivity and specificity, especially in the case of poorly differentiated cells.

In some clinical situations, cellular markers can be used to refine this analysis, but it often remains subjective and limited by the use of light microscopy. Indeed, these imaging tools require cell destruction or selective tagging, thereby negating nonintrusive in vivo and in vitro studies. Fluorescence microscopy (FM), including immunofluorescence microscopy or super-resolution microscopies, exploit selective tagging of proteins/molecules via fluorescent markers to investigate and localize cancer progression in cells or tissues to analyze gene amplification and expression, the morphology of cells and protein localization and dynamics [12]. Therefore, these methods require the specific antibodies or other probes that selectively bind to the component of interest in a cell, and they could interfere with the investigation of the biochemical process itself [5]. Traditional diagnostic imaging tools, including computed tomography scans, magnetic resonance imaging (MRI), positron emission tomography (PET) or ultrasound technology, lack spatial resolution and specificity, showing a high false-positive rate [5,13]. Mass-spectroscopy-based imaging can directly provide the molecular/atomic composition of the sample. However, it is a destructive technology in which samples are dissociated and the relative components are differentiated and interpreted by mass selection [14]. Therefore, there is still a need for novel biophotonic devices allowing rapid, high-throughput metabolic profiling and immunophenotyping at the single-cancer cell level.

Optical spectroscopy approaches have the potential for non- or minimally invasive use in a wide range of clinical applications. Both infrared (IR) and RS have been used in the analyses of biological samples and for the structural characterization of cellular components [15]. Changes in IR and Raman spectra (peak intensity and position) can be easily used to monitor the sub-cellular components of health or diseases cells/tissues, including proteins, lipids, carbohydrates and nucleic acids [15].

In recent years, a combination of confocal systems with IR and Raman has been dedicated to applications involved in the analyses of cells [16]. Compared to the conventional standard histopathology method, IR spectroscopy is frequently applied to analyses of cells due to its low cost of equipment, and speed [15,16,17]. Although IR spectroscopy has already been applied to the analysis of cells, these studies cannot be carried out in living samples due to the strong absorption of water in the infrared region. On the other hand, in Raman spectroscopy analysis, the cell samples can be studied in their physiological environment. Raman spectra can reveal intrinsic biochemical changes that can be used as “Raman signature” to assess physiological status and diagnose disease before any cellular morphological changes can be detected [17], thus enabling early detection of cancer. The minimally invasive nature of RM and the use of endogenous contrast (label-free) make it a very attractive and safe technology for in vivo use.

For the cancer cell sensing, RS is more advantageous than FM as it does not require an external contrast agent and works on the principle of Raman scattering, i.e., a vibrational phenomenon involving inelastic scattering [17]. Moreover, the fingerprint region of Raman spectra provides a better understanding of the chemical changes that occur in cancer cells and can deliver the multiplexed detection for a variety of biological targets in increasingly complex media [18]. It is ideal for cancer studies because of its high chemical specificity, lack of need for sample preparation or cell tagging, the non-destructive nature of the analysis, its speed, and the fact that it is not greatly influenced by the presence of water. Raman microscopy allows for imaging the distribution of chemotherapeutic drugs in cells, opening avenues as a non-invasive and label-free technique to investigate pharmacokinetics at the highest possible resolution in living cells [19]. Indeed, the detection of molecular targeted agents and their metabolites in cells/tissues by label-free RS is attractive because dyes or fluorescent labels may be toxic or invasive [19].

Raman molecular imaging is presently limited by poor spatial resolution and a small field of view, rendering the application of the RS approach to the whole-body scanning difficult. However, several groups are working to overcome this limitation [20,21].

The main features of RS for cancer cell sensing, compared with other techniques, are summarized in Table 1.

**Figure 1:**
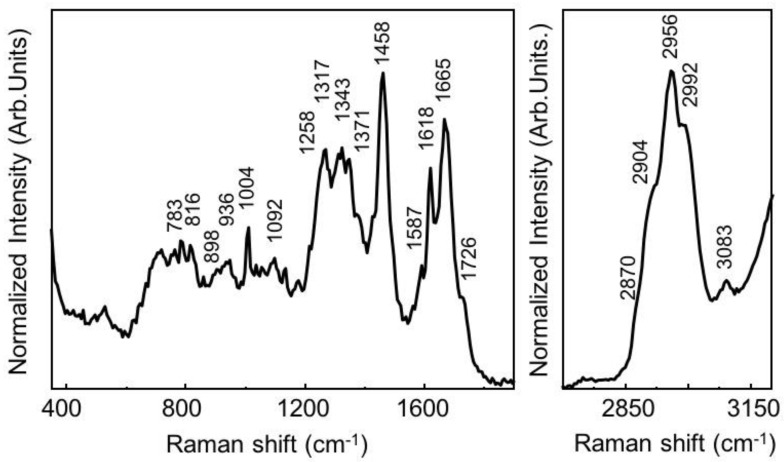
The normalized averaged Raman spectrum of SK-BR3 HER2+ breast cancer cells. The spectral acquisition time was 60 s with 1 accumulation averaged. The green laser with a wavelength of 532 nm was used with 10 mW laser power, 600 grooves/mm grating, 100-micron pinhole and monochromator slit of 50 microns. The Raman spectrum is obtained from our Lab using the measurements of 30 cells averaged.

Figure 1 shows the typical Raman spectrum acquired from SK-BR3 HER2+ breast cancer cells by plotting the intensity of scattered light as a function of frequency. A Raman spectrum is characterized by several bands arising from all the molecules present in the sample and an isolated function group shows a well-known peak location, as shown in Table 2. Indeed, the bands in the spectral region between 600 and 800 cm^−1^ are associated with nucleic acid components. The region between 800 and 1200 cm^−1^ has contributions from DNA backbone, lipids (C-C, C-O stretching), proteins (C-C, C-N stretching), and C-O stretching of carbohydrates. There are several bands ascribed to Amide III, polysaccharides, lipids, and nucleic acids in the region between 1200 and 1400 cm^−1^. The bands in the region between 1400 and 1500 cm^−1^ are essentially due to C-H, CH_2_, and CH_3_ vibrations. The region 1500–1760 cm^−1^ is characterized by the contribution from C=O stretching vibrations (amide I band) with contributions of water, proteins (C=C), nucleic acids, and lipids (C=C stretch). The spectral region between 2700 and 3100 cm^−1^ is essentially ascribed to membrane lipid and phospholipids (C-H, CH_2_, and CH_3_ vibrations). However, the actual peak location of a functional group in a molecule may differ (shift) from the isolated case because of interactions and bonding with its neighbors [17].

However, cells are heterogeneous structures and their Raman features can depend on the position where spectra are measured. Furthermore, Raman spectra require specialized ‘spectroscopic eyes’ for their precise interpretation, which has not facilitated its practical application in cancer diagnosis. In general, the comparison of Raman spectra between normal versus cancer cells have shown differences in the concentration of biochemical compounds of lipids, nucleic acids and proteins. Such Raman spectral variations and band assignments can make it difficult to differentiate the nature of cell types by the clinician. To accomplish global cell sensing that is effective for cancer diagnosis, the development of an automatic and objective method for discriminating cancer cells without any specialized knowledge of spectroscopy is needed.

Selected Raman bands or, alternatively, whole-spectrum analyses by machine learning techniques can be used for the objective identification and discrimination of cells. Both approaches require the development of robust and automated classification tools and spectra pre-processing, including baseline corrections to remove fluorescence or background noise and normalization procedures [22,23,24].

To differentiate small biochemical variations between normal and cancer cells, RS combined with multivariate analysis methods such as principal component analysis (PCA) or linear discriminate analysis (LDA) can be used to identify the accurate and reliable separation of each cell group. Therefore, clinicians can directly differentiate the cell type using automated RS combined with PCA/LDA, without seeing the Raman spectrum, and he/she is not required to have prior spectroscopy knowledge. Thus, PCA/LDA is becoming a widely used method for the spectral analysis of multi-components samples.

In PCA analysis, the dimensions of the Raman data are decomposed, and reduced into a linear combination of loading vectors (principal components (PCs)). The loading vectors can make an orthonormal coordinate system able to reproduce both the total variable variance with all components and to reproduce the correlations. The scores represent the weight coefficients for loading vectors [7]. The PCA score plots, constructed by plotting the score coefficients in the plane of the PCs, can be used to visualize cell groups. In other words, as a result of the PCA, Raman spectra acquired from normal and cancer cell-lines appear as scores distributed in different regions of the score plot [8], allowing an immediate visualization and differentiation of the cell types. The PCA loading can detect spectral differences in cell states and identify the associated biomolecular changes [4,5,6,7,8].

Recently, a powerful biomolecular component analysis (BCA) algorithm has been demonstrated to provide precise information about the biochemical component analysis of cell compartments, including nucleoli, endoplasmic reticulum, Golgi apparatus, and mitochondria, in both fixed cells and living cells [26,27,28]. The BCA toolbox is a stand-alone software package, consisting of three main blocks: the background processing and subtraction, nonlinear least-squares routine, and the graphic user interface. This software package is suitable not only for studies of macromolecular heterogeneity of cell cultures, but can also be applied for probing the absolute concentrations of proteins, RNA and lipids in individual organelles of live cells by using micro-Raman data [26].

### 2.2. Application of Raman Spectroscopy for Cancer Cell Identification

Thanks to the detection abilities described in Section 2.1, RS has been largely used for the identification of cancer/non-cancer cells, as well as to monitor the stage of cancer levels by visualizing and detecting the DNA/RNA, protein, and lipid structural distribution within the cell [29]. Eventually, RS can be used for early stage cancer diagnosis and therapeutic drug monitoring (TDM) [10,30]. In the forthcoming section, we discuss two examples of RS application for cancer cell sensing in fluids including blood cells (leukemia cells) or liquid biopsy (breast cancer).

#### 2.2.1. Leukemia Cells

The RS has been used to differentiate leukocytes from the peripheral blood samples by detecting the intrinsic biochemical components of the same. [31]. The RM has been exploited to discriminate normal lymphocytic B-cells from three different B-acute lymphoblastic leukemia (B-ALL) cell lines (RS4;11, REH, MN60 cells) by their biochemical characteristics [8]. The RS4;11 and REH cell line are both classified as the L2-blast subtype, which has similar morphology, and thus it is difficult to analyze by standard optical methods such as immunofluorescence and Western blotting analysis. While, MN60 cell line is a more differentiated B-ALL cell type which is classified as the L3-blast (i.e., B-cell leukemia) subtype [32,33,34]. The single-point Raman system was used to study the spectral characteristics of each cell line and to identify the biochemical differences between the B-ALL cells and normal cells.

**Figure 2:**
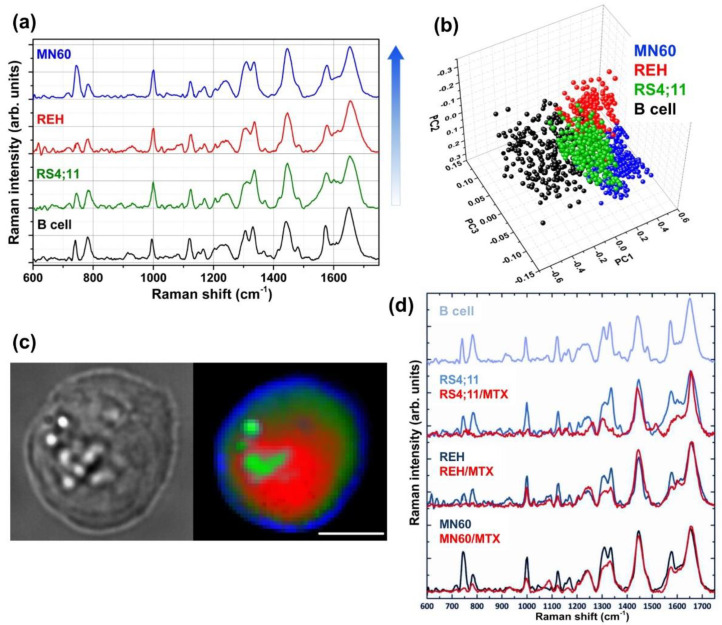
Raman spectroscopic analyses of leukemia cells reveals distinct spectral characteristics for each cell line. (**a**) Average Raman spectra of normal B cells and three B-leukemia cell samples (RS4;11, REH and MN60). The arrow, to the right of the spectra, shows the lymphoblastic B-cell maturation/differentiation stages (The MN60 cell line is the more differentiated). (**b**) PCA scatter plot for the analyzed cell samples. (**c**) White-light optical image and Raman image of an MN60 cell. The colors assigned to the Raman image refer to different Raman signals from different locations within the cells: nucleus (red), cytoplasm (dark green), vesicles (light green) and membrane (blue). The scanning area is 30 × 30 μm^2^ and 45 × 45 pixels; the laser wavelength is 532 nm and the power on the samples is 10 mW. Scale bar: 10 μm. (**d**) Average Raman spectra of B cells and RS4;11, REH and MN60 B-leukemia cell lines before and after the chemotherapic treatment (MTX at 1 mM for 72 h). The Raman spectra are offset for clarity. Reproduced with permission from the Springer Nature publications and Wiley publications, respectively [8,31].

In Figure 2a, the average Raman spectra of normal B-cells and B-ALL cell lines (RS4;11, REH, MN60) are compared and the differences in Raman spectra were used to classify and discriminate between normal and leukemia cell lines. Raman spectra of L2-blast subtype cells (RS4;11 and REH) showed less intense Raman bands at 785, 1120, 1370 and 1577 cm^−1^, which are assigned to the DNA. Such a weak signal is probably due to the breakdown or translocation of chromosomes and reduction in nucleus/cytoplasm ratio [4,8]. In contrast, the Raman spectrum of the MN60 cell lines showed higher intensity of the Raman band around at 745 cm^−1^, which is attributed to cytochrome C, suggesting it is at the most advanced stage of maturation.

On a different note, the Raman spectra of B-ALL cells showed a higher intensity of the Raman amide III (at 1252 cm^−1^), amide I (at 1660 cm^−1^) and CH/CC region bands (at 1337, 1420–1485, 1607–1617 cm^−1^) in comparison to the normal B-cells. This increased Raman bands appeared to be more pronounced from RS4;11 to MN60 cells and confirm a good correlation with the differentiation/maturation stages of these B-leukemia cells.

In order to achieve the quality of cell sensing in terms of sensitivity and specificity, RS was combined with PCA, which provides a good separation between the representative clusters of each cell line (Figure 2b). In particular, (i) the normal B cell cluster shows a larger spread, probably because these cells were collected from different donors; (ii) MN60 cells were completely separated from the other leukemia clusters, confirming a more differentiated cell type. A discrimination efficiency of up to 98% was obtained by combining RS with the statistical method and demonstrating its potential to discriminate normal and cancer cells, particularly in different differentiation/maturation stages. The white-light optical image and Raman imaging of MN60 cell line are additionally reported in Figure 2c.

Preliminary Raman results of three clinical samples have shown that the Raman approach is promising for medical applications. The RS study allowed identification of leukemia cells with a diagnostic sensitivity and specificity up to 87 and 85%, respectively [8]. Basically, distinctive differences in the Raman spectra between normal and cancer cells were obtained, which further reinforced the clinical diagnosis.

In the clinical assessment of a leukemic patient, first step is the analysis and classification of leukemia cells; the second step is to monitor the cells after a specific chemotherapy treatment. The Raman spectral changes in B-ALL cell lines were observed after a 72 h pharmacological treatment using two different chemotherapy drugs such as Methotrexate (MTX) and 6-Mercaptopurine (6-MP) which are able to induce a B-ALL regression, without a cytotoxic effect on cells [8,31]. All-Trans-Retinoic Acid (ATRA) was used as control drug and it is generally used for acute promyelocytic leukemia treatment where the drug is unable to induce B-ALL regression.

After MTX treatment, the B-ALL cells showed a dose-dependent decrease in nucleic acids (at 785, 1120, 1370, and 1577 cm^−1^) and protein contents (at 1004, 1300–1350 and 1400–1500 cm^−1^) (Figure 2d) [31]. The PCA and the confusion matrix showed a clear separation between normal B cells and the MTX-treated B-ALL cells, with a total efficiency of classification above 95%. After 6 MP treatment, B-ALL cells showed lower intensities of the Raman bands (nucleic acids, phenylalanine and proteins) than MTX-treated cells. On the contrary, the ATRA-treated leukemia cells did not exhibit important variations in the spectral region related to the protein–DNA ratio (at 1447 and 785 cm^−1^).

#### 2.2.2. Breast Cancer Cells

RS is particularly suited for sensing breast cancer because of its sensitivity in detecting biochemical component changes that are associated with cancer, such as changes in lipid and protein levels [35]. According to a report, 30% of breast cancers have an amplification of the human epidermal growth factor receptor 2 (HER2)/neu gene or overexpression of HER2 protein [36]. The breast cancer cell lines with various expression levels of the HER2/neu receptor protein such as MDA-MB-231 with HER2–, MDA-MB-435s with moderate HER2+, and SK-BR-3 with HER2+ overexpression was studied using RS [37,38]. To improve the Raman sensing of breast cancer cells differentiation, the normalized average Raman spectra of those cell lines were examined and compared. The MDA-MB-231 and MDA-MB-435s cells showed spread morphologies, while the SK-BR-3 cell population contained both round cells and spread cells. The Raman spectra showed a pronounced increase in lipid expression in both MDA-MB-231 and MDA-MB-435s cell lines when compared to SK-BR-3 cell lines. The strong positive differences (at 1265, 1298, 1440, 1656–1660 cm^−1^) suggested an increased unsaturated fatty acid content in MDA-MB cell lines compared to SK-BR-3 cell lines. The spectral bands at 1265 and 1298 cm^−1^ are assigned to the =CH− in-plane deformation and in-phase −CH_2_ twisting motion, respectively.

The resulting differences in Raman spectra suggest a lower level of fatty acids (lipids) in MDA-MB-231 cells compared to MDA-MB-435s. These changes were observed in both the fingerprint range (1265–1298 cm^−1^) and in the high-frequency Raman range (between 2700–3100 cm^−1^), that reflects the differences in lipid to protein distributions for the different cells [37,38].

X. Bi et al. [39] have shown the feasibility of RS to differentially identify the amplification of HER2 in breast cancer cells. Three breast cancer cell lines, including HER2 protein overexpressing breast cancer cell (BT474), HER2− control (MCF-10A), and HER2+ control (MCF-10A/HER2), were investigated by RM. The difference in Raman spectra were calculated by subtracting from HER2+ control from HER2− control, as well as BT474 from HER2− control and HER2+ control. The positive differences at both the 752 and 1088 cm^−1^ bands are assigned to the increased nucleic acid content which corresponds to the overexpression of HER2 protein in BT474 cells. The strong negative amide III (at 1247, 1270/1278 band ratio and 1438 cm^−1^) Raman bands suggest a decreased HER2 amplification when control (HER2− control and HER2+ control) cell spectra were subtracted from that of BT474 cancer cells. When compared with MCF-10A, MCF-10A/HER2 exhibits an increased level of phosphate and lipids content, but lower than in BT474 cell. Such a dominance of phosphate arises from the enhanced phosphorylation in the HER2-amplified cells.

Recently, the biochemical components of the nuclei (cell nucleus) and lipid droplets (cytoplasm) in the breast cancer cells (MCF-7 and MDA-MB-231) were analyzed and the results were compared with control (MCF-10A) [40]. First, the individual single cell lipid droplets were quantified, and the result showed a 4-fold increase in lipid droplets in the MDA-MB-231 cells than normal, and 2-fold higher than that of MCF7 cells. The increased amount of lipid droplets correlates with the increased aggressiveness of cancer. Second, the Raman single-point spectrum acquired within the nuclei and lipid droplet region of the cells was analyzed. The Raman spectrum of lipid droplets provides information about triacylglycerols, while that of the nucleoli inside the nucleus provides information about proteins. The symmetric stretching terminal −CH_3_ group from the nuclei is markedly blue-shifted for the cancer cells (at 2936 and 2939 cm^−1^ for the MCF7 and MDA-MB-231 cells, respectively) when compared to control (at 2933 cm^−1^). Therefore, the degree of blue shift can also be treated as a Raman signature to discriminate cancer cells from normal. In contrast, the C-H stretching modes of the methylene CH_2_ vibration band at 2852 cm^−1^ are assigned to the fatty acids/triglycerides of the lipid droplets, which do not show any blue shift with increasing aggressiveness of cancer [40].

RS has shown spectral differences between single cells as well as groups of different cells within a cell line, however, they are very small compared to spectral differences between normal and cancer cells. Moreover, RS combined with the PCA method was attempted to visualize and extract useful information from multivariate spectral data to examine qualitative differences among all types of samples.

### 2.3. Raman Microscopy for Cell Imaging and Study of Drug Delivery

Raman imaging rather than single-point spectral analysis could benefit surgeons, allowing more complete visualization of the tumor samples. In a spectroscopic image, the sample is scanned and Raman intensities at the light frequencies specific to molecular markers are mapped as a function of the spatial coordinates of the sample. This allows an assessment of the chemical heterogeneity of a specimen in terms of the spatial distribution of the molecular constituents, providing a false-color map of the sample. The fact that Raman spectroscopy can provide molecular/chemical information of the cell of interest makes it a competitive contender in the molecular imaging arena. Aside from chemical specificity, Raman spectroscopy also possesses many other desirable properties for imaging applications, such as high spatial resolution, high multiplexing capability and excellent photostability [10].

The histochemical staining technique is the standard method for analyzing the cellular morphology such as size, shape and granularity, however, it suffers from some limitations and is not able to provide reliable information on the biochemical changes in or the specific immunophenotype of cells. To overcome these obstacles, Raman imaging can be exploited, allowing the detection of morphological features and the biochemical variations in the cells, which enables the achievement of high classification efficiency in leukemia cells.

The morphology of B-ALL MN60 cells has been studied based on Raman imaging (see Figure 2c), which shows the round-shaped cells with 10–15 µm size, the presence of vacuoles/granules and irregular shaped nucleus [8]. The Raman image of a MN60 leukemia cell was produced with a raster scanning of the cell (15 × 15 μm^2^) using a 532 nm laser and collecting a Raman spectrum for each pixel. A false-color Raman image of the cell was constructed by acquiring about 2500 spectra in about 30 min, which clearly showed the main cellular compartments of the cells, such as nucleus, cytoplasm and vesicles. The Raman spectrum of nucleus shows that bands at 730, 780, 1120, 1370 and 1578 cm^−1^ are attributed to the nucleic acids and the bands at 788 and 1095 cm^−1^ are assigned to the O-P-O backbone. The Raman spectrum of cytoplasm shows a high content of proteins and carbohydrates, which appear, to a lesser extent, between 1300–1350 and 1400–1500 cm^−1^. At last, the Raman spectrum of vesicles reveals intense Raman bands assigned to lipid contents (at 1449, 1250 and 1660 cm^−1^), in particular to vibrations in the hydrocarbon chains.

Besides, the hyperspectral Raman imaging of cells can obtain the morphological information in combination with PCA/LDA analysis, which could improve the identification and classification of different cell phenotypes and lead to non-invasive and accurate monitoring of maturation stages of the disease.

**Figure 3:**
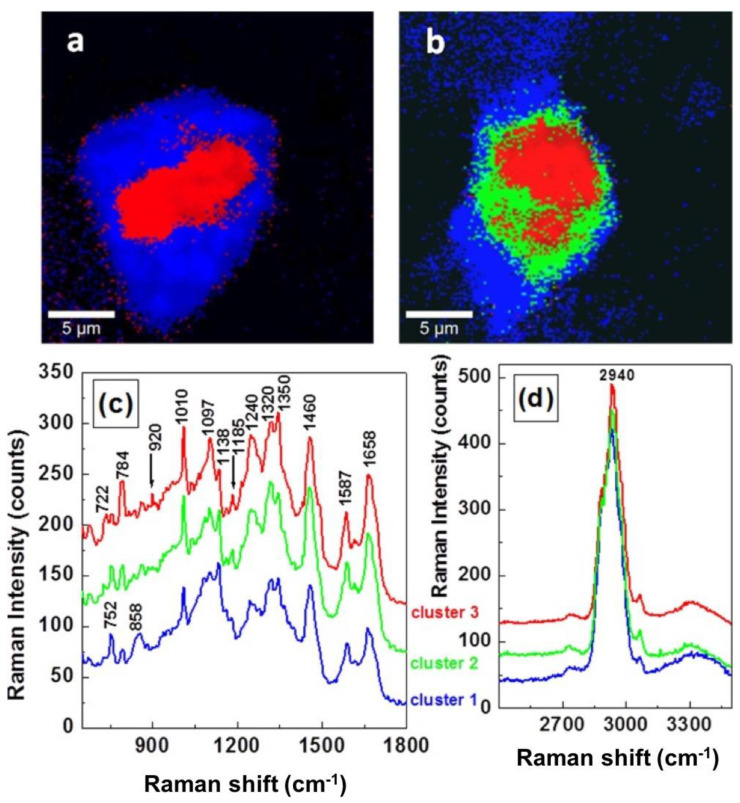
Raman mapping images obtained by Cluster Analysis software of (**a**) untreated MCF-7 cells and (**b**) EGF-treated MCF-7 breast cancer cells, respectively. The individual normalized averaged Raman spectra associated with each cluster with fingerprint region (**c**) and in higher wavenumber region (**d**). The same color code is maintained for images and for spectra. Reproduced with permission from the SAGE publications [41].

Raman imaging has been used to monitor breast tumor signatures. In general, human epidermal growth factor receptor 1 (EGFR or erbB1) sensing in the breast cancer cells is directly achieved by RM, which is responsible for HER2+ overexpression [41]. The Raman images were constructed (using multivariate processing methods) using untreated MCF-7 and MCF-7 cells treated with EGF (well known to contribute to cell proliferation). EGF-treated MCF-7 cells show an obvious increase in the red region, about 80%, when compared to untreated MCF-7 cells, which are only about 20%, as shown in Figure 3a,b. In the blue spectrum, the band at 752 cm^−1^ is dominant, while the intensity of this band starts decreasing in the green spectrum, whereas the band at 784 cm^−1^ starts increasing its contribution.

The intensities of these two bands (at 752 and 784 cm^−1^) are reversed in the red spectrum. Two new bands around at 920 and 1185 cm^−1^ emerged in both the red and green spectrum (marked with arrows in Figure 3c) which are attributed to the existence of phosphorylated threonine and serine, respectively. Such new bands suggest that a phosphorylation process could be the potential reason for the faster cell proliferation of EGF-treated cells. Besides this, a small increase can be seen in the intensity of band at 1350 cm^−1^ in the red spectrum, once more suggesting a structural DNA modification. There are no vibrational changes observed in Figure 3d.

Thanks to its label-free and non-destructive nature, Raman imaging can be successfully applied to non-invasively image living human cancer cells and to study the internalization kinetics and localization of drug nanovectors [42]. The properties of functionalized nanoparticles and nanovectors has been studied extensively and have made it possible to develop new strategies for reducing toxicity to healthy tissues and cells and improving the therapeutic efficiency. The cell uptake and distribution of nanovectors have been routinely analyzed by confocal fluorescence microscopy and transmission electron microscopy (TEM). However, fluorescence labels could modify the size and chemical properties of the vector, perturbing the study of their intracellular delivery and properties. On the other hand, TEM imaging offers high-resolution images down to the nanoscale, but it is a time-consuming and destructive technique.

Of note, Raman imaging can be used to study the uptake and the localization of silica-based diatomite nanoparticles (DNPs, size around 300–400 nm) conjugated with a non-targeting siRNA into cancer cell lines (H1355) for up to 72 h [42].

**Figure 4:**
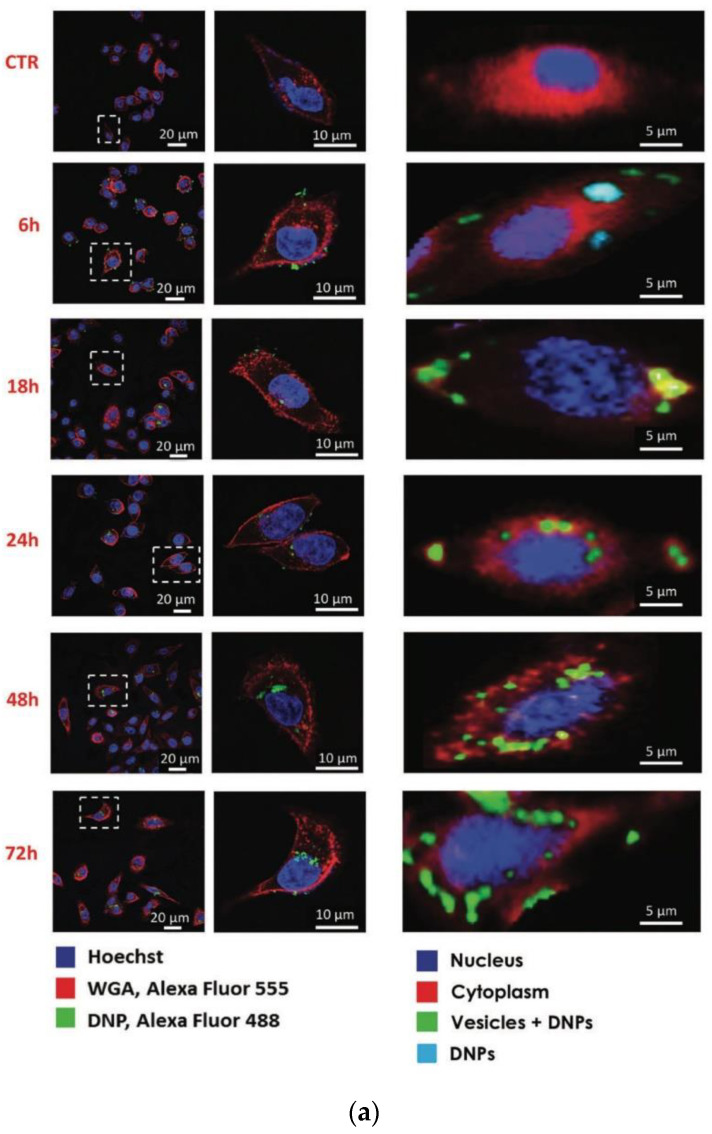

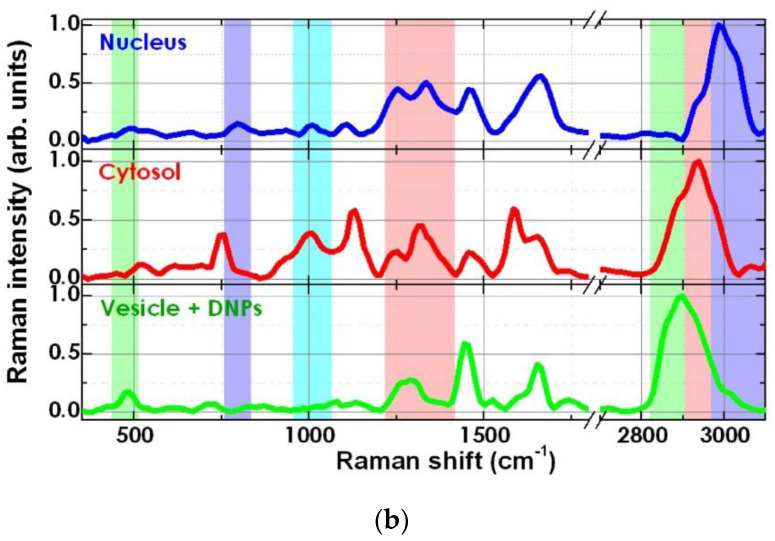
(**a**) Confocal (left) and Raman imaging (right) of DNP uptake in H1355 cells at different incubation times (0, 6, 18, 24, 48 and 72 h). (**b**) MCR-ALS spectra corresponding to nucleus (blue), cytosol (red), vesicle + DNPs (green). Reproduced with permission from the Wiley publications [42].

RM does not require labelling, and therefore cellular uptake of the DNPs can be probed without chemical/charge alterations in their surface. The RM results were compared with confocal fluorescence analysis (Figure 4a). Each Raman image was obtained by raster scanning the cell with a laser at 532 nm and recording a 2D array with a step size of 0.5 μm of Raman spectra on a selected area (about 2000 spectra for each image) [42]. Each Raman spectrum was acquired with an integration time of 0.5 s and background-corrected using a polynomial fitting approach. The multivariate curve resolution–alternative least square (MCR-ALS) method was used to obtain false-colour Raman images. This method is in-house algorithm, which generates loadings similar to spectra, which are easier to chemically/physically code. The spectra/loadings of different components of the cells were converted into spatial distribution maps and translated into brightness pixels in the cell image. For each step, three or more cells were randomly analyzed, and the imaging analysis was repeated three times. In Figure 4b, MCR-ALS spectra corresponding to nucleus (blue), cytosol (red), vesicle + DNPs (green) are shown [42].

The Raman biochemical signature combined with the spatial information allowed to locate the DNPs (band below 600 cm^−1^) inside the lipid vesicles (bands in the range 2800–3000 cm^−1^), in the perinuclear region of cytoplasm, and to monitor the uptake dynamics and kinetics of siRNA + DNPs complex in the cells for up to 72 h. Indeed, the fate of DNPs is relevant to their cytotoxicity, while their intracellular persistence is crucial to increase the effective dose of transported drugs. On the other hand, the FM imaging provided ambiguous conclusions about the environment in which the DNPs are located within the cells, and it was unclear form the fluorescence-based imaging study if the nanovectors remained attached to the cell membrane or internalized.

The analytical capabilities of RM are not restricted to specific cell types and/or nanocarriers and could be extended to other drug-delivery systems. However, Raman imaging experiments require the acquisition of many thousands of spectra and, typically, total acquisition times for these, depending on the desired spectral quality, would be in the order of a few minutes to several hours. Recently, the application of spatial light modulators (SLM) in RS has been demonstrated to create multifoci or light scanning for excitation of Raman scattering [43,44] or for a combination of structured illumination with compressive detection to improve the acquisition speed of Raman imaging [45].

Surface-enhanced Raman scattering (SERS) is typically implemented in place of, or in combination with, RS to address the key limitations of such technique [46]. In this regard, recently, Wang et al. described a topical-staining protocol for rapid and quantitative multiplex SERS imaging of cancer biomarkers on the surfaces of freshly resected tissues to be used as an intraoperative tool for surgical guidance [46,47].

### 2.4. High-Throughput Raman Cell Sorting

A key obstacle for RS applications in cancer diagnosis is the acquisition time for single spectra (from the fraction of seconds to even minutes), resulting in small data sizes (generally less than hundred cells are analyzed) and questionable statistical significance of the data [48]. SERS has been extensively used to enhance the signals from biomolecules, but it requires the development of specific SERS substrates and/or functionalized metallic nanoparticles [49,50].

RM combined with a microfluidic device, called the Raman-activated cell sorting (RACS) platform has been proposed for the fast diagnosis and selection of circulating tumor cells and for functional sorting of cells [51]. However, the weak Raman signals greatly limit the advantages of RACS systems to achieve the high throughput in single-cell sorting. This can be improved using advanced Raman spectrometers to achieve efficiency in Raman-acquisition and integration of the RACS system into the novel microfluidic devices. Such a modified RACS system has been shown to achieve high-throughput cell sorting, which can reach an analytical rate of several to dozens of cells per second.

Popp et al. have recently proposed a high-throughput screening RS (HTS-RS) platform based on the combination of an automated bright-field recognition routine able to select the single-cell focal plane with Raman spectroscopy for rapid and label-free macromolecular fingerprinting of tens of thousands of eukaryotic cells [52].

**Figure 5:**
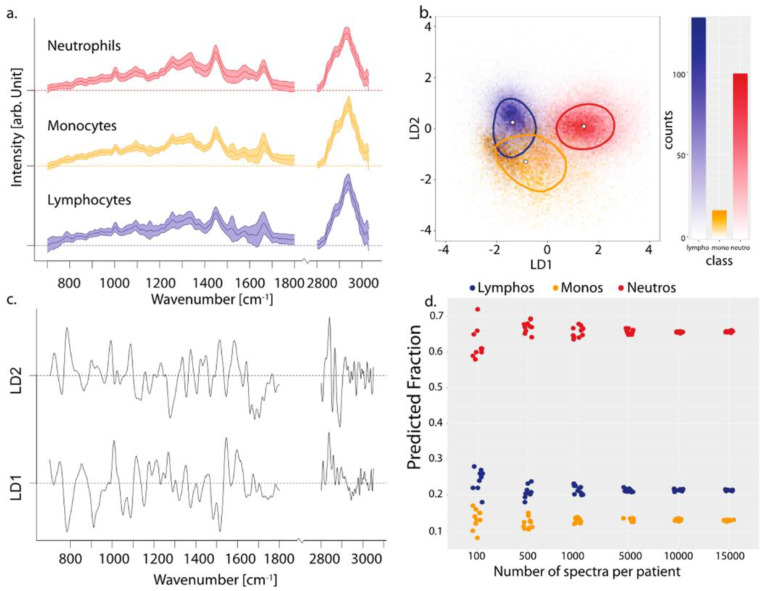
(**a**) Mean and standard deviation of normalized Raman spectra of three cell classes. (**b**) Three cell spectra projected onto space spanned by LD1 and LD2. The median of each group is shown by the white dot and the line indicates the 50th percentile. The LD1 and LD2 scattering plot model shows measurements of a total of 107,738 cells. (**c**) The corresponding linear discriminant analysis (LDA) coefficients for LD1 and LD2. (**d**) Random drawing of various sample sizes. Reproduced with permission from the American Chemical Society [52].

Three major leukocyte types, such as neutrophils, lymphocytes, and monocytes were isolated from four healthy volunteers to establish a model for the identification of cell types in mixed populations by HTS-RS combined with partial least-squares-linear discriminant analysis (PLS-LDA) classification models. For this study, the total number of acquired Raman spectra for four volunteers was 107,732, with a maximum acquisition speed of up to 20,000 cells in less than 4 h. The results, summarized in Figure 5, reveal three completely separated cell clusters.

Three different ratios of cancer cell/leucocytes 50/50, 1/10, and 1/100 were additionally studied. All measurements were performed in an automated fashion with a developed data acquisition method. It was shown that, even for the lowest mixture, i.e., 1/100, the prediction based on the HTS-RS measurements provides highly accurate results, and the sampling of nearly 1000 cells could be achieved in 38 min. On a different note, the HTS-RS approach was truly valuing the advantage of RS; because a minimal sample preparation is required and large, statistically significant sample sizes in a mixed population, removes the human dependency from the data acquisition and a substantial amount of data can be accumulated for an explicit statistical evaluation.

A similar experimental set-up was made based on a high-content analysis Raman spectroscopy (HCA-RS) platform to the development of a Raman-based cell viability assay, in terms of the effect of doxorubicin (DOX) concentration on monocytic THP-1 cells as monocytic acute myeloid leukemia cell line [53]. The drug-induced changes in cells were then investigated by HCA-RS, with, in total, 25,031 cells sampled to differentiate between viable and non-viable cells by PCA combined with support vector machine (PCA-SVM). PCA-SVM was applied to evaluate the percentage of viable cells in a mixed population and the method is further verified with standard cell viability assay. Changes in several Raman bands, such as nucleic acid, protein and lipid contents, are related to apoptosis, leading to DNA denaturation, cessation of DNA replication and DNA intercalation in cancer cells compared with control cells.

The first two principal components (PC1 and PC2) were used to build the SVM model, which constitute 95.65% of the total spectral variance in the dataset. The first and the second PCs correspond to 74.26% and 21.39% of the spectral variance, respectively. Therefore, the PCA-SVM model was used to estimate the percentage of cell viabilities in the mixed population of all other test conditions. This meant that the future development of the HCA-RS would include real-time living cells, cell type assignment and cell sorting using Raman spectral information.

The ability of the HTS and HCA-RS platform to probe the biochemical differentiates of a large number of cells, more than 1000 cells within 30 min, and such newly developed technique has demonstrated the potential of RS as an all-around tool for cell studies, eventually paving the way for biomedical and clinical applications.

### 2.5. Correlative Raman with Other Microscopies for Cancer Cell Sensing

The required RS optical apparatus is built around a conventional microscope, and hence the technology can be developed as an adjunct to routine microscopy. RM can be combined with other optical techniques, for example, FM, optical coherence tomography (OCT), digital holography and mass spectroscopy-based imaging, providing an integrated and multiplexed optical imaging system.

Raman imaging alone does not allow live, high-resolution imaging of large areas in complex matrices, for instance in tissues, where the cells and extracellular matrix coexist and interact. To overcome this limitation, wide-field fluorescence imaging was performed on a confocal Raman microscope. The methodology developed allowed for unprecedented chemical characterization of fluorescently labeled biological tissues in vivo [54].

Great advantages would be gained by combining RS with morphological approaches such as OCT or digital holography. By combing RS and OCT, it is possible to provide a more complete description of the analyzed sample, correlating the biochemical information of the Raman analysis with microstructural and morphological information from the OCT. In 2008, Anita Mahadevan-Jansen et al. presented, for the first time, a dual-mode RS-OCT device allowing the detection of the Raman signal and the OCT image through common sampling optics, while keeping the excitation source and detection separated [55]. The proposed device was able to compensate for the limitations of the two techniques alone. Basically, OCT performed real-time, large-area imaging of the tissues and was used to guide the Raman analysis. Single-point Raman spectra were acquired to specifically characterize the biochemical fingerprint of ambiguous structures within the OCT tissue image [51]. Pilot experiments by Liu et al. demonstrated successful proof-of-concept tumor margin prediction in near-clinical situations by the OCT-Raman system and potential as a novel optical biopsy technique for cancer detection [56].

Shape, morphological defects and optical thickness are also useful parameters, particularly for the discrimination between cells, and may be recorded via holography. Digital Holographic Microscopy (DHM) is label-free microscopy based on the interference pattern created by two coherent beams. An algorithm allows the 3D reconstruction of the object (e.g., a cell) that caused the pattern [57]. The great advantage of digital holography is the possibility of retrieving 3D quantitative imaging of the sample under investigation with a single-shot acquisition and directly in its native environment [7]. The technique is fast and has unique imaging capabilities for time-lapse investigations on both the single-cell and the cell-population levels. It also requires very low light intensity, thus rendering the approach free from phototoxicity. It has been demonstrated that RS and DHM can be used simultaneously to provide a morphological and biochemical analysis of cells [58] and it can be applied to living white blood cells [59]. The phase map can provide information on the shape, dimensions and presence of cell defects that can be correlated with the biochemical information provided by single-point Raman spectra of the analyzed cells [58]. Holography has a much faster acquisition time than RS, which may provide a fast initial screening, and the specific molecular analysis can be performed on selected cells. The two approaches can validate each other for a more robust analysis [59].

When using two orthogonally polarized reference waves, it possible to reconstruct not only morphology information but also intrinsic information about the polarization state of the sample through the phase change quantification [60]. Indeed, it has been observed that cells/tissues are composed of regularly arranged protein filaments that may affect their birefringence behavior [61]. Therefore, polarization-sensitive digital holographic imaging (PSDHI) combined with RS can be used to correlate information on morphology, birefringence and biochemical composition for a complete analysis of the cell sample.

Raman imaging excels at measuring cellular states in a non-invasive and non-destructive manner, which makes it useful for screening and assessing drug response at the single-cell level [42]. However, it is complicated to quantify the drug uptake or metabolism response at a single cell level using the RM alone. Recently, it has been demonstrated that correlating the Raman signal with the destructive mass spectrometry analysis renders the drug and metabolite quantification at a single-cell level possible [62]. Indeed, the combined platform is capable of providing more information about drug uptake, metabolism, and effects at the single-cell level, demonstrating the potential to investigate pharmacokinetics at the single-cell level.

## 3. Summary and Future Perspective

The present review showcases a few important aspects/potentials of RS and RM, which range from molecular detection to diagnosis. In this context, we analyzed two examples of leukemia and breast cancer cells using RS and RM that shown the possibility of label-free, non-invasive identification of cancer cells and classification. However, in clinical practice, these techniques cannot be used by pathologists and clinicians. The translation for clinical usage involves the development of advanced automatic spectrometer and objective methods for discriminating the cancer cells without any specialized knowledge of spectroscopy, such as PCA or LDA. These automated statistical approaches, using large numbers of spectra, may also identify molecular patterns among cancer types, become predictors of the aggressiveness of cancer (cancer grading) and allow for follow-up after chemotherapy treatment.

Indeed, RS was used for the specific cell identification and classifications of leukemia cells based on intrinsic Raman markers associated with DNA, proteins and lipids and that allowed identification of biochemical differences between acute lymphoblastic leukemia type B (B-ALL) cell lines (RS4;11, REH and MN60) and normal cells with a high accuracy of 98% [8,31].

Similarly, RS and RM have shown that Raman spectral differences between normal cells and breast cancer cells were more pronounced. The breast cancer cells tend to have significantly less lipid content than a normal cell, whereas cancer cells have shown increased nucleic acid content, which corresponds to the overexpression of HER2 protein (HER2+) [38]. It was also found that an increased number of lipid droplets are present in breast cancer cells, which suggested an increasing aggressiveness of cancer.

Raman mapping rather than single-point spectral analysis could benefit surgeons, allowing a more complete visualization of the cancer cells. As most pathologists and other clinicians are not spectroscopically well-trained, RS will be useful to them if there is a way to present the thousands of Raman signals, obtained from different cells, in an easily interpretable format, i.e., a Raman map. Indeed, Raman imaging has shown the potential to follow the internalization kinetics and localization of drug nanovectors in the cells [42]. RS and RM have shown the ability to measure the spatial distribution of proteins, lipids and nucleic acids in cancer cells with high chemical specificity, submicrometric resolution, high multiplexing capability and excellent photostability. Because of these unique properties, combined with an exceptional information depth, RS and RM are ideal for label-free high-throughput and high-content cell screening.

A key limitation for RS applications in cancer diagnosis is the long acquisition time required for single spectra, resulting in small data sizes and questionable statistical significance of the data. This issue can be further improved by enhancing the Raman signal, as in the SERS experiments, or by integrating advanced Raman spectrometers into microfluidic devices [51] or using recently developed high-throughput screening RS (HTS-RS) [52].

Integrated and multiplexed optical imaging systems, obtained by combining RS with either OCT or digital holography, can provide fast and large area imaging of tissue/cell sample. This combination can be used for correlating the single-point Raman analysis information of biochemical changes in the cells with microstructural and morphological information. These approaches have demonstrated successful proof-of-concept tumor margin prediction in near-clinical conditions within their native environment.

However, the transfer of the “Raman spectral library”, such as those available in FTIR analyses, remains a major challenge that stands in the way of clinical translation for Raman-based technologies, and continued effort is needed to facilitate the transition from benchtop to bedside by developing novel experimental designs and structured protocols for the objective method. The further development of the cell classification methods, the improvement in Raman instrumentations trending toward obtaining data with greater resolution, shorter collection times, and higher accuracy would give additional information, allowing earlier and more accurate diagnosis, reducing the need for human intervention, and increasing the objectivity of assessment [11,63].

## Figures and Tables

**Table 1:** Raman spectroscopy (RS) of cancer cells sensing compared with other techniques.

	FTIR	MRI	MSI	H&E	FM	RS
Sample preparation	No	Yes	Yes	Yes	Yes	No
Spatial resolution	>1 μm	>1 mm	Cellular	Sub-cellular	>200 nm	>0.5 μm
Destructive	No	No	Yes	No	No	No
Contrast agents	No	No	No	Yes	Yes	No
Water interference	Yes	No	No	No	No	No
Real-time	Yes	No	No	No	Yes	Yes
Miniature, portable-handy	Yes	No	No	Yes	No	Yes
Multiplexing	Yes	No	Yes	No	No	Yes

FM—Fluorescence microscopy; MSI—Mass spectroscopy-based imaging; H&E—Hematoxylin and Eosin stain; MRI—Magnetic resonance imaging; FTIR—Fourier transform infrared spectroscopy; RS—Raman spectroscopy.

**Table 2:** Raman spectroscopy band assignments of SK-BR3 breast cancer cells.

Raman Shift (cm^−1^)	Assignment [25]
783	Cytosine U, C, T ring breathing, DNA (nucleic acids assignment)
816	Proline, tyrosine, PO^2−^ stretching (nucleic acids assignment)
898	Glycans, polysaccharides (β-glucose), (C-O-C) skeletal mode
936	C-C backbone (collagen assignment)
1004	C-C Symmetric stretching ring breathing, phenylalanine (protein assignment)
1092	P=O symmetric vibration from nucleic acids/cell membrane phospholipids (protein assignment)
1258	Amide III, adenine, cytosine (protein assignment)
1317	C-H deformation (lipid/protein assignment)
1343	C-H deformation of proteins (protein assignment)
1371	The most pronounced saccharide band, Ring and C-N stretch
1458	Fatty acids, triglycerides, CH_2_ bending or CH_2_/CH_3_ deformation of (lipids and collagen assignment)
1587	Amide II, aromatic amino acids within proteins of C=C olefinic stretch (protein assignment)
1618	C=C Symmetric stretching, tryptophan and phenylalanine (protein assignment)
1665	Amide I, Unsaturated fatty acids, C=O stretching (C-H) deformation/(C=C) stretch (lipid assignment)
1726	C=O Symmetric stretching; glycans and glycogen (lipid assignment)
2870	CH_3_ stretching vibrations, lipids, fatty acids, Amide I (lipid assignment)
2904	CH_2_ antisymmetric stretching, CH_3_ symmetric stretching (lipid assignment)
2956	CH_3_ asymmetric stretch (protein assignment)
2992	combination of ring stretching vibrations of CH_2_ and CH (lipids/protein assignment)
3083	C–H aromatic stretching (nucleic acids/proteins assignment)
